# Design and Analysis Methods for Trials with AI-Based Diagnostic Devices for Breast Cancer

**DOI:** 10.3390/jpm11111150

**Published:** 2021-11-04

**Authors:** Lu Liu, Kevin J. Parker, Sin-Ho Jung

**Affiliations:** 1 Department of Biostatistics and Bioinformatics, Duke University, Durham, NC 27710, USA; lu.liu@duke.edu; 2 Department of Electrical and Computer Engineering, University of Rochester, Rochester, NY 14627, USA; kevin.parker@rochester.edu

**Keywords:** artificial intelligence (AI), breast cancer, clinical device trial, concordance rate, generalized estimating equation, sample size calculation, statistical test

## Abstract

Imaging is important in cancer diagnostics. It takes a long period of medical training and clinical experience for radiologists to be able to accurately interpret diagnostic images. With the advance of big data analysis, machine learning and AI-based devices are currently under development and taking a role in imaging diagnostics. If an AI-based imaging device can read the image as accurately as experienced radiologists, it may be able to help radiologists increase the accuracy of their reading and manage their workloads. In this paper, we consider two potential study objectives of a clinical trial to evaluate an AI-based device for breast cancer diagnosis by comparing its concordance with human radiologists. We propose statistical design and analysis methods for each study objective. Extensive numerical studies are conducted to show that the proposed statistical testing methods control the type I error rate accurately and the design methods provide required sample sizes with statistical powers close to pre-specified nominal levels. The proposed methods were successfully used to design and analyze a real device trial.

## 1. Introduction

There are different types of device trials depending on the use of device and the study objectives. In this paper, we introduce statistical design and analysis methods for a trial on an artificial intelligence (AI)-based device for the diagnosis of breast cancer.

Imaging technologies play a major role in the diagnosis of breast cancer. The reading and interpretation of imaging requires intensive medical training and significant clinical experience. With the advance of big data analysis methods, machine learning and AI-based imaging systems are currently under active development [1]. If an AI-based imaging device can read the image as accurately as experienced radiologists, it may be able to help radiologists increase the accuracy of their reading, manage their workloads, or possibly replace radiologists in remote clinics that would not have an experienced radiologist available for consultation.

In the assessment of breast lesions, the BI-RADS reporting system and classification are widely used [2]. This system includes categories between 1 and 5 (benign to malignant) with a key diagnostic transition subdivided into categories 4a (low suspicion of malignancy), 4b (moderate suspicion) and 4c (high suspicion, greater than 50% likelihood but less than 95% likelihood of malignancy). Furthermore, the BI-RADS lexicon covers radiological descriptive features that are important in diagnostic assessments, and these vary by modality. Examples of ultrasound lexicon used in AI-based classifications is given in Table A1. The earlier approaches to breast ultrasound technology concentrated on the extraction of features of lesions such as size, shape, texture, and boundaries within a clustering or classification or rule-based decision making algorithms [3,4,5,6]. More recent developments in AI, machine learning, and deep learning systems have utilized layers of convolution neural network models, a variety of approaches and extensive training sets to produce differentiated output classifications [7,8].

In this paper, we consider the requirements for a clinical device trial to evaluate the performance of an AI-based imaging device using BI-RADS reporting system for the diagnosis of breast cancer. Since BI-RADS reporting system does not have a gold standard, we evaluate the performance of the device by how well its reading aligns with those of radiologists. We propose design and analysis methods for two different types of study objectives that can be used for such a trial. The first objective is to test if the reading of the AI-based device concurs with those of radiologists as much as the readings concord among radiologists. The second objective is to test if the reading of the AI-based device is more concordant with those of experienced radiologists than with those of junior radiologists. For each objective, we propose a statistical testing method and its sample size calculation formula. The proposed testing methods will be used to analyze the data for each of the five BI-RADS lexicon classification category listed in Table A1, but the sample size calculation for a trial may be conducted only for the most important one. The performance of these methods are evaluated using simulations.

## 2. Materials and Methods

We consider two types of study objectives to evaluate the performance of an AI-based device for the diagnosis of breast cancer. For each study objective, we propose a testing method and its sample size formula. Suppose that we have images from *n* subjects.

### 2.1. Objective 1: Is the Concordance Rate between the AI-Based Device and Radiologists as High as That among Radiologists?

The image of each subject is read by *m* radiologists and the AI-based device. BI-RADS lexicon does not have a gold standard. So, in order to validate a device with an AI-based algorithm, we should show that the reading of the device concurs with those with radiologists. For example, in Table A1, for BI-RADS lexicon classification, Shape, two radiologists will be declared to be concurrent for an image if they both read oval, round or irregular. The question is how high the concordance rate should be between the device and the radiologists. The concordance rate among radiologists is used as a reference.

For each category of BI-RADS lexicon classifications, let $p_{r}$ and $p_{s}$ denote the concordance rate among radiologists and that between radiologists and the device, respectively. Since the latter can not be higher than the former, we specify a similarity margin $\delta_{1}\left(>0\right)$. That is, we will not be interested in the AI-based device if $p_{s}\leq p_{r}-\delta_{1}$ and will be highly interested in it if $p_{s}=p_{r}$. So, we want to test a null hypothesis $H_{1}:p_{s}=p_{r}-\delta_{1}$ against the alternative hypothesis ${\bar{H}}_{1}:p_{s}>p_{r}-\delta_{1}$.

#### 2.1.1. Statistical Testing Method

Suppose that there are *n* patients, and the image of each patient is read by the AI-based device and *m* radiologists. For subject i(=1,…,n) and radiologist j(=1,…,m), let $r_{ijj^{\prime }}=1$ if radiologists *j* and $j^{\prime }$ concur and =0 otherwise, and let $s_{ij}=1$ if radiologist *j* and the device concur and =0 otherwise. Note that we have $p_{r}=E\left(r_{ijj^{\prime }}\right)$ and $p_{s}=E\left(s_{ij}\right)$. Since $r_{ijj^{\prime }}=r_{ij^{\prime }j}$ and $r_{ijj}=1$ for $j,j^{\prime }=1,\ldots ,m$, the number of informative concordance scores among *m* radiologists is m(m−1)/2 for each image, the concordance rate among radiologists for subject *i* is estimated by

$$r_{i}=\frac{\sum_{j=1}^{m-1}\sum_{j^{\prime }=j+1}^{m}r_{ijj^{\prime }}}{m(m-1)/2}.$$

On the other hand, for subject *i*, the concordance rate between the device and *m* radiologists is estimated by

$$s_{i}=\frac{\sum_{j=1}^{m}s_{ij}}{m}$$

Using the images from *n* subjects, concordance rate among radiologists is estimated by

$${\hat{p}}_{r}=\frac{2}{nm(m-1)}\sum_{i=1}^{n}\sum_{j=1}^{m-1}\sum_{j^{\prime }=j+1}^{m}r_{ijj^{\prime }}=\frac{1}{n}\sum_{i=1}^{n}r_{i}$$

and that between the device and radiologists is estimated by

$${\hat{p}}_{s}=\frac{1}{nm}\sum_{i=1}^{n}\sum_{j=1}^{m}s_{ij}=\frac{1}{n}\sum_{i=1}^{n}s_{i}$$

Those estimates are unbiased because

$$E\left({\hat{p}}_{r}\right)=E\left(\frac{2}{nm(m-1)}\sum_{i=1}^{n}\sum_{j=1}^{m-1}\sum_{j^{\prime }=j+1}^{m}r_{ijj^{\prime }}\right)=\frac{2}{nm(m-1)}\frac{nm(m-1)}{2}p_{r}=p_{r}$$

and

$$E\left({\hat{p}}_{s}\right)=E\left(\frac{1}{nm}\sum_{i=1}^{n}\sum_{j=1}^{m}s_{ij}\right)=\frac{1}{nm}nmp_{s}=p_{s}.$$

Since $r_{1},\ldots ,r_{n}$ are independent random variables with mean $p_{r}$, for large *n* by the central limit theorem,

$$\sqrt{n}\left({\hat{p}}_{r}-p_{r}\right)=\frac{1}{\sqrt{n}}\sum_{i=1}^{n}\left(r_{i}-p_{r}\right)$$

is asymptotically normal with mean 0 and variance $\sigma_{r}^{2}$ that can be consistently estimated by

$${\hat{\sigma }}_{r}^{2}=\frac{1}{n}\sum_{i=1}^{n}{\left(r_{i}-{\hat{p}}_{r}\right)}^{2}$$

Similarly, $s_{1},\ldots ,s_{n}$ are independent random variables with mean $p_{s}$, so that for large *n*,

$$\sqrt{n}\left({\hat{p}}_{s}-p_{s}\right)=\frac{1}{\sqrt{n}}\sum_{i=1}^{n}\left(s_{i}-p_{s}\right)$$

is asymptotically normal with mean 0 and variance $\sigma_{s}^{2}$ that can be consistently estimated by

$${\hat{\sigma }}_{s}^{2}=\frac{1}{n}\sum_{i=1}^{n}{\left(s_{i}-{\hat{p}}_{s}\right)}^{2}$$

Since each subject’s image is read by the device and radiologists, $r_{i}$ and $s_{i}$ are correlated. However, $\left(s_{i}-r_{i}+\delta_{1},i=1,\ldots ,n\right)$ are independent, with mean 0 under the null hypothesis $H_{1}$. Hence, by the central limit theorem under $H_{1}$,

$$\sqrt{n}\left({\hat{p}}_{s}-{\hat{p}}_{r}+\delta_{1}\right)=\frac{1}{\sqrt{n}}\sum_{i=1}^{n}\left(s_{i}-r_{i}+\delta_{1}\right)$$

is asymptotically normal with mean 0 and variance $\sigma_{1}^{2}$ that can be consistently estimated by

$${\hat{\sigma }}_{1}^{2}=\frac{1}{n}\sum_{i=1}^{n}{\left(s_{i}-r_{i}+\delta_{1}\right)}^{2}$$

Hence, we reject the null hypothesis $H_{1}:p_{s}\leq p_{r}-\delta_{1}$ if $Z_{1}>z_{1-\alpha }$, where

$$Z_{1}=\frac{\sqrt{n}\left({\hat{p}}_{s}-{\hat{p}}_{r}+\delta_{1}\right)}{{\hat{\sigma }}_{1}}$$

and $z_{1-\alpha }$ is the 100(1−α) percentile of the standard normal distribution. Note that we use a 1-sided test because the hypotheses are 1-sided and to avoid too large a sample size with a small $\delta_{1}$.

Note that ${\hat{p}}_{r}$ and ${\hat{p}}_{s}$ are the generalized estimating Equation [9] (GEE) estimators of $p_{r}$ and $p_{s}$, respectively, using the working independent correlation. Furthermore, the robust estimator of $\sigma_{1}^{2}$ by the GEE method is given as

$${\tilde{\sigma }}_{1}^{2}=\frac{1}{n}\sum_{i=1}^{n}{\left(s_{i}-r_{i}+{\hat{p}}_{s}-{\hat{p}}_{r}\right)}^{2}$$

Since ${\hat{p}}_{s}-{\hat{p}}_{r}$ is a consistent estimator of $p_{s}-p_{r}$, ${\tilde{\sigma }}_{1}^{2}$ is asymptotically identical to ${\hat{\sigma }}_{1}^{2}$ under $H_{1}$. Hence, $Z_{1}$ can be counted as a test statistic based on the GEE method with the working independent correlation.

#### 2.1.2. Power and Sample Size Calculation

We calculate the sample size for the test statistic $Z_{1}$ under a specific alternative hypothesis ${\bar{H}}_{1}:p_{r}=p_{s}$. An accurate sample size calculation for the statistical test requires specification of correlation coefficients between $r_{ij_{1}j_{2}}$ and $r_{ij_{1}^{\prime }j_{2}^{\prime }}$, between $r_{ij_{1}j_{2}}$ and $s_{ij}$, and between $s_{ij}$ and $s_{ij^{\prime }}$. The dependency between $r_{ij_{1}j_{2}}$ and $r_{ij_{1}j_{2}^{\prime }}$ is expected to be higher than that between $r_{ij_{1}j_{2}}$ and $r_{ij_{1}^{\prime }j_{2}^{\prime }}$ for $j_{1}\neq j_{1}^{\prime }\neq j_{2}\neq j_{2}^{\prime }$ because the former pair includes the same reader $j_{1}$ while the latter pair contains four different readers. Similarly, we expect that the dependency between $r_{ij_{1}j_{2}}$ and $s_{ij_{1}}$ is expected to be higher than that between $r_{ij_{1}j_{2}}$ and $s_{ij_{1}^{\prime }}$ for $j_{1},j_{2}\neq j_{1}^{\prime }$.

For a simplified sample size formula, we just specify $\rho_{1}=\mathrm{corr}\left(r_{i},s_{i}\right)$. We define the correlation coefficients among the concordance scores $\rho_{r1}=\mathrm{corr}\left(r_{i12},r_{i13}\right)$, $\rho_{r2}=\mathrm{corr}\left(r_{i12},r_{i34}\right)$, $\rho_{s1}=\mathrm{corr}\left(r_{i12},s_{i1}\right)=\mathrm{corr}\left(r_{i12},s_{i2}\right)$, and $\rho_{s2}=\mathrm{corr}\left(r_{i12},s_{i3}\right)$, and $\rho_{ss}=\mathrm{corr}\left(s_{i1},s_{i2}\right)$. Appendix A.1 shows that we have $\rho_{1}=\mathrm{corr}\left(r_{i},s_{i}\right)$ is expressed as

$$\rho_{1}=\frac{\frac{2}{m}\rho_{s1}+\frac{m-2}{m}\rho_{s2}}{\sqrt{\left(\frac{2}{m(m-1)}+\frac{4(m-2)}{m(m-1)}\rho_{r1}+\frac{\left(m-2)(m-3\right)}{m(m-1)}\rho_{r2}\right)\left(\frac{1}{m}+\frac{m-1}{m}\rho_{ss}\right)}}$$

Under ${\bar{H}}_{1}:p_{r}=p_{s}$, $\left\{\left(s_{i}-r_{i}\right),i=1,\ldots ,n\right\}$ are independent random variables with mean 0, so that $\sqrt{n}\left({\hat{p}}_{s}-{\hat{p}}_{r}\right)$ is asymptotically normal with mean 0 and variance $\sigma_{1}^{2}$ that can be consistently estimated by $s_{1}^{2}={\sqrt{n}}^{-1}\sum_{i=1}^{n}{\left(s_{i}-r_{i}\right)}^{2}$. Since ${\hat{\sigma }}_{1}^{2}$ is asymptotically identical to $s_{1}^{2}+\delta_{1}^{2}$ under ${\bar{H}}_{1}$, it converges to $\sigma_{1}^{2}+\delta_{1}^{2}$. Hence, the power for a given sample size *n* is

$$1-\beta =P\left(\frac{\sqrt{n}\left({\hat{p}}_{s}-{\hat{p}}_{r}+\delta_{1}\right)}{{\hat{\sigma }}_{1}}>z_{1-\alpha }|p_{r}=p_{s}\right)$$

$$=P\left(\frac{\sqrt{n}\left({\hat{p}}_{s}-{\hat{p}}_{r}\right)+\sqrt{n}\delta_{1})}{\sqrt{\sigma_{1}^{2}+\delta_{1}^{2}}}>z_{1-\alpha }|p_{r}=p_{s}\right)$$

$$=P\left(\frac{\sqrt{n}\left({\hat{p}}_{s}-{\hat{p}}_{r}\right)}{\sigma_{1}}>\frac{z_{1-\alpha }\sqrt{\sigma_{1}^{2}+\delta_{1}^{2}}-\sqrt{n}\delta_{1}}{\sigma_{1}}|p_{r}=p_{s}\right)$$

(1) $$=\Phi \left(\frac{z_{1-\alpha }\sqrt{\sigma_{1}^{2}+\delta_{1}^{2}}-\sqrt{n}\delta_{1}}{\sigma_{1}}\right)\;$$

where Φ(.) is the survivor function of the standard normal distribution and $\sigma_{1}^{2}$ is the limit of $n^{-1}\sum_{i=1}^{n}{\left(s_{i}-r_{i}\right)}^{2}$.

By solving the power Equation (1) with respect to *n*, we obtain the required sample size for power 1−β

(2) $$n=\frac{{\left(z_{1-\alpha }\sqrt{\sigma_{1}^{2}+\delta_{1}^{2}}+z_{1-\beta }\sigma_{1}\right)}^{2}}{\delta_{1}^{2}}\;$$

where, as shown in the Appendix A.2,

(3) $$\sigma_{1}^{2}=\mathrm{var}\left(s_{i}\right)+\mathrm{var}\left(r_{i}\right)-2\rho_{1}\sqrt{\mathrm{var}\left(s_{i}\right)\mathrm{var}\left(r_{i}\right)}\;$$

$$\mathrm{var}\left(s_{i}\right)=p_{r}\left(1-p_{r}\right)\left\{1/m+\rho_{ss}\left(m-1\right)/m\right\}$$

and

$$\mathrm{var}\left(r_{i}\right)=p_{r}\left(1-p_{r}\right)\left(\frac{2}{m(m-1)}+\frac{4(m-2)}{m(m-1)}\rho_{r1}+\frac{\left(m-2)(m-3\right)}{m(m-1)}\rho_{r2}\right).$$

The process of calculating the required sample size is summarized as follows:

(1) Specify (α,1−β), expected concordance rate among radiologists $p_{r}$, similarity margin $\delta_{1}$ and hypothetical correlation coefficients $\rho_{r1},\rho_{r2},\rho_{ss},\rho_{s1}$ and $\rho_{s2}$.
(2) Calculate $\sigma_{1}^{2}$ using (3).
(3) Obtain sample size using (2).

It may be difficult to specify the correlation coefficients $\rho_{r1},\rho_{r2},\rho_{ss},\rho_{s1}$ and $\rho_{s2}$. If pilot data are available, we may estimate them from the pilot data. Otherwise, we may conduct a two-stage trial to estimate these correlation coefficients from the first stage data and recalculate the sample size for the whole trial based on the estimated correlation coefficients.

### 2.2. Objective 2: Is the AI-Based Device More Concordant with Experienced Radiologists Than with Junior Radiologists?

As another study objective, we may want to test if the reading of the AI-based device agrees more with those of experienced radiologists than with those of junior radiologists for each BI-RADS lexicon classification category.

Let $p_{x}$ and $p_{y}$ denote the concordance rate between the AI-based device and highly experienced radiologists and that between the AI-based device and less experienced radiologists, respectively. We want to test the null hypothesis $H_{2}:p_{x}=p_{y}$ against the alternative hypothesis ${\bar{H}}_{2}:p_{x}>p_{y}$.

#### 2.2.1. Statistical Testing Method

Let *m* (= 5, say) denote the number of radiologists in each group (highly experienced group and less experienced group). For subject i(=1,…,n) and senior radiologist j(=1,…,m), let $x_{ij}=1$ if the reading by senior radiologist *j* and that by the AI-based device agree and =0 otherwise, and let $y_{ij}=1$ if the reading by less experienced radiologist j(=1,…,m) and that by the AI-based device agree and =0 otherwise. Then, we have $p_{x}=E\left(x_{ij}\right)$ and $p_{y}=E\left(y_{ij}\right)$. Using the data from subject *i*,, $p_{x}$ is estimated by

$$x_{i}=\frac{\sum_{j=1}^{m}x_{ij}}{m}$$

and $p_{y}$ is estimated by

$$y_{i}=\frac{\sum_{j=1}^{m}y_{ij}}{m}$$

Using the whole data, we estimate $p_{x}$ and $p_{y}$ by

$${\hat{p}}_{x}=\frac{1}{nm}\sum_{i=1}^{n}\sum_{j=1}^{m}x_{ij}=\frac{1}{n}\sum_{i=1}^{n}x_{i}$$

and

$${\hat{p}}_{y}=\frac{1}{nm}\sum_{i=1}^{n}\sum_{j=1}^{m}y_{ij}=\frac{1}{n}\sum_{i=1}^{n}y_{i}$$

respectively. Note that those estimates are unbiased.

For large *n* under $H_{2}$, $\sqrt{n}\left({\hat{p}}_{x}-{\hat{p}}_{y}\right)$ is approximately normal with mean 0 and variance $\sigma_{2}^{2}$ that can be estimated by

$${\hat{\sigma_{2}}}^{2}=\frac{1}{n}\sum_{i=1}^{n}{\left(x_{i}-y_{i}\right)}^{2}$$

Hence, we reject $H_{2}:p_{x}=p_{y}$ if $|Z_{2}|>z_{1-\alpha /2}$, where

$$Z_{2}=\frac{\sqrt{n}\left({\hat{p}}_{x}-{\hat{p}}_{y}\right)}{{\hat{\sigma }}_{2}}.$$

Note that we use a standard 2-sided test since usually there is no small effect size issue in this case.

#### 2.2.2. Power and Sample Size Calculation

We calculate the sample size under a specific alternative hypothesis ${\bar{H}}_{2}:p_{x}=p_{y}+\delta_{2}$. Since each subject’s image is read by all experienced and inexperienced radiologists as well as the device, $\left\{\left(x_{ij},y_{ij}\right),j=1,\ldots ,m\right\}$ are correlated.

Let $\rho_{xx}=\mathrm{corr}\left(x_{i1},x_{i2}\right)$ denote the correlation coefficient between the concordance score between the AI-based device and a highly experienced radiologist and the concordance score between the device and another highly experienced radiologist, and $\rho_{yy}=\mathrm{corr}\left(y_{i1},y_{i2}\right)$ denote the correlation coefficient between the AI-based device and a less experienced radiologist and the concordance score between the device and another less experienced radiologist. Furthermore, let $\rho_{xy}=\mathrm{corr}\left(x_{ij},y_{ij^{\prime }}\right)$ for $j,j^{\prime }=1,\ldots ,m$ denote the correlation coefficient between the concordance score between the device and a highly experienced radiologist and that between the device and a less experienced radiologist. Let $\rho_{2}=\mathrm{corr}\left(x_{i},y_{i}\right)$. As shown in Appendix A.3. $\rho_{2}$ is a function of $\rho_{xx},\rho_{yy}$, and $\rho_{xy}$.

Under ${\bar{H}}_{2}:p_{x}=p_{y}+\delta_{2}$, $\left\{\left(x_{i}-y_{i}-\delta_{2}\right),i=1,\ldots ,n\right\}$ are independent random variables with mean 0, so that $\sqrt{n}\left({\hat{p}}_{x}-{\hat{p}}_{y}-\delta_{2}\right)$ is asymptotically normal with mean 0 and variance $\sigma_{2}^{2}$ that can be consistently estimated by $s_{2}^{2}={\sqrt{n}}^{-1}\sum_{i=1}^{n}{\left(x_{i}-y_{i}-\delta_{2}\right)}^{2}$. Note that ${\hat{\sigma }}_{2}^{2}$ is asymptotically identical to $s_{2}^{2}+\delta_{2}^{2}$ under ${\bar{H}}_{2}$, so that it converges to $\sigma_{2}^{2}+\delta_{2}^{2}$. Hence, the power for a given sample size *n* is

$$1-\beta =P\left(\frac{\sqrt{n}\left({\hat{p}}_{x}-{\hat{p}}_{y}\right)}{{\hat{\sigma }}_{2}}>z_{1-\alpha /2}|p_{x}=p_{y}+\delta_{2}\right)$$

$$=P\left(\frac{\sqrt{n}\left({\hat{p}}_{x}-{\hat{p}}_{y}-\delta_{2}\right)+\sqrt{n}\delta_{2}}{\sqrt{s_{2}+\delta_{2}^{2}}}>z_{1-\alpha /2}|p_{x}=p_{y}+\delta_{2}\right)$$

$$=P\left(\frac{\sqrt{n}\left({\hat{p}}_{x}-{\hat{p}}_{y}-\delta_{2}\right)}{\sigma_{2}}>\frac{z_{1-\alpha /2}\sqrt{\sigma_{2}^{2}+\delta_{2}^{2}}-\sqrt{n}\delta_{2}}{\sigma_{2}}|p_{x}=p_{y}+\delta_{2}\right)$$

(4) $$=\Phi \left(\frac{z_{1-\alpha /2}\sqrt{\sigma_{2}^{2}+\delta_{2}^{2}}-\sqrt{n}\delta_{2}}{\sigma_{2}}\right)\;$$

since $\sqrt{n}\left({\hat{p}}_{x}-{\hat{p}}_{y}-\delta_{2}\right)/\sigma_{2}$ is N(0,1) under ${\bar{H}}_{2}$, where $\sigma_{2}^{2}$ is the limit of $n^{-1}\sum_{i=1}^{n}{\left(x_{i}-y_{i}-\delta_{2}\right)}^{2}$ under ${\bar{H}}_{2}$.

By solving (4) with respect to *n*, we obtain the required sample size for power 1−β

(5) $$n=\frac{{\left(z_{1-\alpha /2}\sqrt{\sigma_{2}^{2}+\delta_{2}^{2}}+z_{1-\beta }\sigma_{2}\right)}^{2}}{\delta_{2}^{2}}\;$$

Appendix A.4 shows that

(6) $$\sigma_{2}^{2}=\mathrm{var}\left(x_{i}\right)+\mathrm{var}\left(y_{i}\right)-2\rho_{2}\sqrt{\mathrm{var}\left(x_{i}\right)\mathrm{var}\left(y_{i}\right)}\;$$

where

$$\mathrm{var}\left(x_{i}\right)=p_{x}\left(1-p_{x}\right)\left(\frac{1}{m}+\frac{m-1}{m}\rho_{xx}\right)$$

and

$$\mathrm{var}\left(y_{i}\right)=\left(p_{x}-\delta_{2}\right)\left(1-p_{x}+\delta_{2}\right)\left(\frac{1}{m}+\frac{m-1}{m}\rho_{yy}\right)$$

under ${\bar{H}}_{2}$.

The process of calculating the required sample size is summarized as follows:

1. Specify (α,1−β), expected concordance rate between the AI-based device and a highly experienced radiologist $p_{x}$, clinically meaningful difference in concordance rates $\delta_{2}$ and correlation coefficients $\rho_{xx},\rho_{yy}$ and $\rho_{xy}$.
2. Calculate $\sigma_{2}^{2}$ using (6).
3. Obtain the required sample size using (5).

It will be difficult to specify the correlation coefficients $\rho_{xx},\rho_{yy}$ and $\rho_{xy}$. If pilot data are available, we may estimate them from the pilot data. Otherwise, we may use a two-stage design to estimate these correlation coefficients from the first stage data and calculate the sample size for the whole trial based on the estimated correlation coefficients.

## 3. Numerical Studies and Results

Note that our test statistics and sample size formulas are derived based on large sample approximations. We want to conduct simulation studies to evaluate their finite sample performance.

We consider the first type of study objective to test if the concordance rate between an AI-based device and radiologists is as high as that among radiologists. Suppose that each subject’s image is read by the AI-based device and m=10 radiologists. We set α=0.05, 1−β=0.8 or 0.9, $p_{r}=0.3,\;0.5\;\text{or}\;0.7$, $\delta_{1}=0.05\;\text{or}\;0.1$ and $\rho_{1}=0.1,\;0.3,\;0.5,\;\text{or}\;0.7$. Assuming $\rho_{s1}=\rho_{s2}+0.1,\rho_{r1}=\rho_{r2}+0.1,\rho_{r1}=\rho_{ss}=\rho_{s1}+0.1$, we calculate the correlation coefficients for a given $\rho =\mathrm{corr}(r_{i},s_{i})$. That is, we obtain $\left(\rho_{s1},\rho_{s2},\rho_{ss},\rho_{r1},\rho_{r2}\right)=\left(0.101,0.001,0.201,0.201,0.101\right)$, (0.16,0.06,0.26,0.26,0.16), (0.26,0.16,0.36,0.36,0.26) and (0.48,0.38,0.58,0.58,0.48) for $\rho_{1}=0.1,\;0.3,\;0.5,\;\text{or}\;0.7$, respectively.

For each design setting, we calculate the required sample size *n* using our proposed formula (2) and generate 10,000 simulation data sets of size *n* under the design setting and $H_{1}$ or ${\bar{H}}_{1}$. Then, we apply the statistical testing using $Z_{1}$ to each simulation data set, and compute the empirical type I error ($\hat{\alpha }$) and power ($1-\hat{\beta }$) by the proportion of samples that reject $H_{1}$ among the 10,000 samples simulated under $H_{1}:p_{s}=p_{r}-\delta_{1}$ and ${\bar{H}}_{1}:p_{s}=p_{r}$, respectively. The correlated concordance (binary) data are generated by first generating multivariate normal data and then dichotomizing them with corresponding proportion level [10].

Table 1 reports the sample size *n*, empirical type I error rate $\hat{\alpha }$, and power $1-\hat{\beta }$ under each design setting. We observe that the required sample size increases in 1−β and decreases in $\delta_{1}$ and $\rho_{1}$. With other design parameters fixed, we have the same sample sizes for $p_{r}=0.3$ and $p_{r}=0.7$. We have this result because, from (2), the sample size depends on $p_{r}$ only through $p_{r}\left(1-p_{r}\right)$. Since the empirical type I errors are very close to the nominal α=0.05 overall, our test statistic $Z_{1}$ controls the type I error rate accurately. On the other hand, the empirical powers are close to the corresponding nominal level 1−β=0.8 or 0.9 overall, so that we conclude that our sample size formula is accurate too.

Now we conduct simulations for the second type of study objective to test if the AI-based device is more concordant with experienced radiologists than with junior radiologists. We assume that each subject’s image is read by m=5 experienced radiologists and m=5 junior radiologists. We set α=0.05, 1−β=0.8 or 0.9, $p_{x}=0.3,\;0.5,\;\text{or}\;0.7$, $\delta_{2}=0.05\;\text{or}\;0.1$, and $\rho_{2}=0.1,\;0.3,\;0.5,\;\text{or}\;0.7$. Assuming $\rho_{xx}=\rho_{yy}=\rho_{xy}+0.1$, we solve the corresponding correlation coefficients for given $\rho_{2}=\mathrm{corr}\left(x_{i},y_{i}\right)$. So, we have $\left(\rho_{xx},\rho_{yy},\rho_{xy}\right)=\left(0.13,0.13,0.03\right)$, (0.21,0.21,0.11), (0.33,0.33,0.23), and (0.55,0.55,0.45) for $\rho_{2}=0.1,\;0.3,\;0.5$, and 0.7, respectively. For each design setting, we calculate sample size *n* using (5), and generate 10,000 samples of size *n* under the design setting and $H_{2}$ or ${\bar{H}}_{2}$. We apply the test statistic $Z_{2}$ to each sample, and calculate the empirical type I error rate and power $\left(\hat{\alpha },1-\hat{\beta }\right)$ under $H_{2}:p_{x}=p_{y}$ and ${\bar{H}}_{2}:p_{x}=p_{y}+\delta_{0}$, respectively.

Table 2 summarizes the required sample size *n*, and empirical type I error rate and power, $\left(\hat{\alpha },1-\hat{\beta }\right)$, under each design setting. We observe that the required sample size increases in 1−β and decreases in $\delta_{2}$ and $\rho_{2}$. Since the empirical type I errors are very close to the nominal α=0.05 overall, our test statistic $Z_{2}$ controls the type I error accurately. On the other hand, the empirical powers are close to the corresponding nominal level 1−β=0.8 or 0.9 overall, so that we conclude that our sample size formula is accurate too.

## 4. Discussion and Conclusions

Existing papers on comparing correlated concordance rates mainly focus on comparing two (or more) competitive diagnosis methods using their concordance rates with a gold standard on multiple sites [11]. In this paper, there is no gold standard and we compare the concordance rate between an AI-based diagnostic device and human radiologists and that among radiologists. We also compare the concordance rate between an AI-based diagnostic device and highly experienced radiologists and that between AI-based device and less experienced radiologists. In our design setting, each study subject has single site but is rated by the AI-based device and multiple human radiologists. We extend existing methods to perform design and analysis in this new setting.

We provide design and analysis plan for two types of study objectives to perform different comparisons of concordance between the AI-based diagnostic device and human radiologists. For each type of study objective, we propose a test statistic using GEE method with independent working correlation to account for the dependency in the observations from the device and the radiologists for each study subject, and derive its sample size formula based on large sample theory. Through extensive simulations, we show that the test statistics control the type I error accurately and the sample size formulas estimate sample sizes with powers close to the specified ones accounting for the dependency of images read by radiologists and device.

Since each subject’s image is read by the device and many radiologists, the concordance scores have complicated dependency structure, while the test statistics do not require specification of the multiple correlation coefficients by using the GEE method, the sample size formulas require specification of these correlation coefficients. Since it is difficult to accurately specify the correlation coefficients, we propose to conduct a two-stage device trial to estimate these correlation coefficients from the first stage data and recalculate the required sample size for the whole trial based on the estimated correlation coefficients.

We use concordance rate as a measure of agreement among multiple raters. Cohen’s kappa is another measure of agreement that is popularly used, e.g., Qureshi et al. [12]. Unlike concordance rate, however, it is not clear how similar two kappa values should be to conclude similarity of two different groups of raters. The proposed methods were successfully used by O’Connell et al. [8] to design and analyze a device trial.

## Figures and Tables

**Table 1:** Sample size (empirical type I error rate, empirical power), $n(\hat{\alpha },1-\hat{\beta })$, under various design settings of ($p_{r},\delta_{1},\rho_{1},1-\beta$) for the first type of study objective.

$p_{r}$	$\delta_{1}$	$\rho_{1}$	1−β=0.8	1−β=0.9
0.3	0.05	0.1	210(0.044,0.808)	290(0.051,0.910)
		0.3	206(0.048,0.812)	285(0.047,0.903)
		0.5	200(0.049,0.805)	275(0.049,0.910)
		0.7	186(0.054,0.811)	256(0.056,0.903)
	0.1	0.1	56(0.041,0.829)	76(0.045,0.915)
		0.3	55(0.047,0.823)	75(0.042,0.914)
		0.5	53(0.048,0.822)	73(0.052,0.921)
		0.7	50(0.061,0.822)	68(0.060,0.913)
0.5	0.05	0.1	249(0.047,0.804)	344(0.048,0.904)
		0.3	245(0.051,0.808)	338(0.053,0.901)
		0.5	237(0.045,0.812)	327(0.050,0.907)
		0.7	220(0.053,0.798)	304(0.050,0.904)
	0.1	0.1	66(0.052,0.815)	90(0.048,0.911)
		0.3	65(0.049,0.824)	88(0.046,0.914)
		0.5	63(0.051,0.831)	86(0.048,0.912)
		0.7	58(0.054,0.813)	80(0.054,0.909)
0.7	0.05	0.1	210(0.052,0.804)	290(0.054,0.902)
		0.3	206(0.050,0.800)	285(0.048,0.906)
		0.5	200(0.052,0.802)	275(0.051,0.906)
		0.7	186(0.055,0.806)	256(0.049,0.899)
	0.1	0.1	56(0.055,0.821)	76(0.054,0.909)
		0.3	55(0.055,0.816)	75(0.049,0.904)
		0.5	53(0.052,0.814)	73(0.054,0.911)
		0.7	50(0.060,0.821)	68(0.058,0.912)

**Table 2:** Sample size (empirical type I error rate, empirical power), $n(\hat{\alpha },1-\hat{\beta })$, under various design settings of ($p_{x},\delta_{2},\rho_{2},1-\beta$) for the second type of study objective.

$p_{x}$	$\delta_{2}$	$\rho_{2}$	1−β=0.8	1−β=0.9
0.3	0.05	0.1	348(0.052,0.810)	465(0.052,0.898)
		0.3	328(0.050,0.812)	438(0.048,0.894)
		0.5	298(0.049,0.807)	397(0.047,0.900)
		0.7	245(0.053,0.807)	327(0.048,0.907)
	0.1	0.1	86(0.054,0.816)	113(0.050,0.905)
		0.3	81(0.053,0.820)	107(0.047,0.912)
		0.5	74(0.047,0.825)	98(0.047,0.914)
		0.7	63(0.053,0.844)	83(0.048,0.934)
0.5	0.05	0.1	434(0.050,0.798)	580(0.048,0.902)
		0.3	409(0.050,0.810)	546(0.049,0.904)
		0.5	370(0.049,0.806)	495(0.052,0.905)
		0.7	304(0.054,0.802)	406(0.050,0.905)
	0.1	0.1	111(0.053,0.816)	148(0.053,0.909)
		0.3	105(0.047,0.814)	140(0.051,0.909)
		0.5	96(0.050,0.811)	127(0.052,0.911)
		0.7	79(0.052,0.829)	105(0.050,0.913)
0.7	0.05	0.1	382(0.051,0.803)	511(0.052,0.900)
		0.3	360(0.052,0.797)	481(0.048,0.901)
		0.5	327(0.046,0.804)	436(0.055,0.902)
		0.7	269(0.047,0.811)	359(0.053,0.909)
	0.1	0.1	103(0.050,0.815)	136(0.049,0.914)
		0.3	97(0.054,0.817)	129(0.049,0.912)
		0.5	89(0.050,0.821)	117(0.050,0.917)
		0.7	74(0.048,0.840)	98(0.045,0.925)

## Appendix A

**Table A1:** Examples of ultrasound lexicon.

Ground Truth	Lesion Type
Shape	Oval
	Round
	Irregular
Margin	Circumscribed
	Indistinct
	Angular
	Microlobulated
	Spiculated
Orientation	Parallel
	Not parallel
Echo pattern	Anechoic
	Hypoechoic
	Complex cystic and solid
	Isoechoic
	Hyperechoic
	Heterogeneous
Posterior features	No features
	Enhancement
	Shadowing
	Combined pattern

### Appendix A.1. Derivation of ρ_1_

Since $r_{i}={\left\{m\left(m-1\right)/2\right\}}^{-1}\sum_{j=1}^{m-1}\sum_{j^{\prime }=j+1}^{m}r_{ijj^{\prime }}$ and $s_{i}=m^{-1}\sum_{j=1}^{m}s_{ij}$, we have

$$\rho_{1}=\mathrm{corr}\left(r_{i},s_{i}\right)=\frac{\mathrm{cov}(r_{i},s_{i})}{\sqrt{\mathrm{var}\left(r_{i}\right)\mathrm{var}\left(s_{i}\right)}}$$

Here

$$\mathrm{cov}\left(r_{i},s_{i}\right)=\mathrm{cov}\left(\frac{\sum_{j=1}^{m-1}\sum_{j^{\prime }=j+1}^{m}r_{ijj^{\prime }}}{m(m-1)/2},\frac{\sum_{j=1}^{m}s_{ij}}{m}\right)=\frac{2}{m^{2}\left(m-1\right)}\mathrm{cov}\left(\sum_{j=1}^{m-1}\sum_{j^{\prime }=j+1}^{m}r_{ijj^{\prime }},\sum_{j=1}^{m}s_{ij}\right)$$

$$=\frac{2}{m^{2}\left(m-1\right)}\left\{m\left(m-1\right)\mathrm{cov}\left(r_{ij_{1}j_{2}},s_{ij_{1}}\right)+\frac{m(m-1)(m-2)}{2}\mathrm{cov}\left(r_{ij_{1}j_{2}},s_{ij_{1}^{\prime }}\right)\right\}$$

$$=\frac{2}{m}\mathrm{cov}\left(r_{i12},s_{i1}\right)+\frac{m-2}{m}\mathrm{cov}\left(r_{i12},s_{i3}\right)$$

$$\mathrm{var}\left(r_{i}\right)=\frac{4}{m^{2}{\left(m-1\right)}^{2}}\mathrm{var}\left(\sum_{j=1}^{m-1}\sum_{j^{\prime }=j+1}^{m}r_{ijj^{\prime }}\right)$$

$$=\frac{4}{m^{2}{\left(m-1\right)}^{2}}\{\frac{m(m-1)}{2}\mathrm{var}\left(r_{ij_{1}j_{2}}\right)+m\left(m-1\right)\left(m-2\right)\mathrm{cov}\left(r_{ij_{1}j_{2}},r_{ij_{1}j_{2}^{\prime }}\right)$$

$$+\frac{m(m-1)(m-2)(m-3)}{4}\mathrm{cov}\left(r_{ij_{1}j_{2}},r_{ij_{1}^{\prime }j_{2}^{\prime }}\right)\}$$

$$=\frac{2}{m(m-1)}\mathrm{var}\left(r_{i12}\right)+\frac{4(m-2)}{m(m-1)}\mathrm{cov}\left(r_{i12},r_{i13}\right)+\frac{\left(m-2)(m-3\right)}{m(m-1)}\mathrm{cov}\left(r_{i12},r_{i34}\right)$$

and

$$\mathrm{var}\left(s_{i}\right)=\frac{1}{m^{2}}\left\{m\mathrm{var}\left(s_{i1}\right)+m\left(m-1\right)\mathrm{cov}\left(s_{i1},s_{i2}\right)\right\}$$

$$=\frac{1}{m}\mathrm{var}\left(s_{i1}\right)+\frac{m-1}{m}\mathrm{cov}\left(s_{i1},s_{i2}\right)$$

Hence,

$$\rho_{1}=\frac{\frac{2}{m}\mathrm{cov}\left(r_{i12},s_{i1}\right)+\frac{m-2}{m}\mathrm{cov}\left(r_{i12},s_{i3}\right)}{\sqrt{\left\{\frac{2}{m(m-1)}\mathrm{var}\left(r_{i12}\right)+\frac{4(m-2)}{m(m-1)}\mathrm{cov}\left(r_{i12},r_{i13}\right)+\frac{\left(m-2)(m-3\right)}{m(m-1)}\mathrm{cov}\left(r_{i12},r_{i34}\right)\right\}}}$$

$$*\frac{1}{\sqrt{\left\{\frac{1}{m}\mathrm{var}\left(s_{i1}\right)+\frac{m-1}{m}\mathrm{cov}\left(s_{i1},s_{i2}\right)\right\}}}$$

$$=\frac{\frac{2}{m}\rho_{s1}+\frac{m-2}{m}\rho_{s2}}{\sqrt{\left\{\frac{2}{m(m-1)}+\frac{4(m-2)}{m(m-1)}\rho_{r1}+\frac{\left(m-2)(m-3\right)}{m(m-1)}\rho_{r2}\right\}\left(\frac{1}{m}+\frac{m-1}{m}\rho_{ss}\right)}}$$

### Appendix A.2. The Limit of ${\hat{\sigma }}_{1}^{2}$ under ${\bar{H}}_{1}$

The limit of ${\hat{\sigma }}_{1}^{2}=n^{-1}\sum_{i=1}^{n}{\left(s_{i}-r_{i}\right)}^{2}$ is its expected value $\sigma_{1}^{2}=E{\left(s_{i}-r_{i}\right)}^{2}$. Since $E\left(s_{i}-r_{i}\right)=p_{s}-p_{r}=0$ under ${\bar{H}}_{1}:p_{r}=p_{s}$, $E{\left(s_{i}-r_{i}\right)}^{2}=\mathrm{var}\left(s_{i}-r_{i}\right)=\mathrm{var}\left(s_{i}\right)+\mathrm{var}\left(r_{i}\right)-2\rho_{1}\sqrt{\mathrm{var}\left(s_{i}\right)\mathrm{var}\left(r_{i}\right)}$ where $\rho_{1}=\mathrm{corr}\left(r_{i},s_{i}\right)$,

$$\mathrm{var}\left(s_{i}\right)=\frac{1}{m^{2}}\mathrm{var}\left(\sum_{j=1}^{m}s_{ij}\right)=\frac{1}{m^{2}}\left\{m\mathrm{var}\left(s_{ij}\right)+m\left(m-1\right)\mathrm{cov}\left(s_{ij},s_{ij^{\prime }}\right)\right\}$$

$$=\frac{1}{m}\mathrm{var}\left(s_{ij}\right)+\frac{m-1}{m}\rho_{ss}\mathrm{var}\left(s_{ij}\right)=p_{s}\left(1-p_{s}\right)\left(\frac{1}{m}+\frac{m-1}{m}\rho_{ss}\right)$$

and

$$\mathrm{var}\left(r_{i}\right)=\mathrm{var}\left(\frac{1}{m(m-1)/2}\sum_{j_{1}=1}^{m-1}\sum_{j_{2}=j+1}^{m}r_{ij_{1}j_{2}}\right)$$

$$={\left\{\frac{1}{m(m-1)/2}\right\}}^{2}\{\frac{m(m-1)}{2}\mathrm{var}\left(r_{i12}\right)+m\left(m-1\right)\left(m-2\right)\mathrm{cov}\left(r_{i12},r_{i13}\right)$$

$$+\frac{m(m-1)(m-2)(m-3)}{4}\mathrm{cov}\left(r_{i12},r_{i34}\right)\}$$

$$=(\frac{2}{m(m-1)}\mathrm{var}\left(r_{i12}\right)+\frac{4(m-2)}{m(m-1)}\rho_{r1}\mathrm{var}\left(r_{i12}\right)+\frac{\left(m-2)(m-3\right)}{m(m-1)}\rho_{r2}\mathrm{var}\left(r_{i12}\right))$$

$$=p_{r}\left(1-p_{r}\right)\left\{\frac{2}{m(m-1)}+\frac{4(m-2)}{m(m-1)}\rho_{r1}+\frac{\left(m-2)(m-3\right)}{m(m-1)}\rho_{r2}\right\}$$

since $s_{ij}∼\mathrm{Bernoulli}\left(p_{s}\right)$ and $r_{ij_{1}j_{2}}∼\mathrm{Bernoulli}\left(p_{r}\right)$.

### Appendix A.3. Derivation of ρ_2_

Since $x_{i}=m^{-1}\sum_{j=1}^{m}x_{ij}$ and $y_{i}=m^{-1}\sum_{j=1}^{m}y_{ij}$,

$$\rho_{2}=\mathrm{corr}\left(x_{i},y_{i}\right)=\frac{\mathrm{cov}(x_{i},y_{i})}{\sqrt{\mathrm{var}\left(x_{i}\right)\mathrm{var}\left(y_{i}\right)}}$$

Here,

$$\mathrm{cov}\left(x_{i},y_{i}\right)=\mathrm{cov}\left(\frac{\sum_{j=1}^{m}x_{ij}}{m},\frac{\sum_{j=1}^{m}y_{ij}}{m}\right)=\mathrm{cov}\left(x_{ij},y_{ij}\right)$$

$$\mathrm{var}\left(x_{i}\right)=\frac{1}{m}\mathrm{var}\left(x_{i1}\right)+\frac{m-1}{m}\mathrm{cov}\left(x_{i1},x_{i2}\right)$$

and, similarly,

$$\mathrm{var}\left(y_{i}\right)=\frac{1}{m}\mathrm{var}\left(y_{i1}\right)+\frac{m-1}{m}\mathrm{cov}\left(y_{i1},y_{i2}\right)$$

Hence,

$$\rho_{2}=\frac{\mathrm{cov}(x_{i1},y_{i1})}{\sqrt{\left\{\frac{1}{m}\mathrm{var}\left(x_{i1}\right)+\frac{m-1}{m}\mathrm{cov}\left(x_{i1},x_{i2}\right)\right\}\left\{\frac{1}{m}\mathrm{var}\left(y_{i1}\right)+\frac{m-1}{m}\mathrm{cov}\left(y_{i1},y_{i2}\right)\right\}}}$$

$$=\frac{\rho_{xy}}{\sqrt{\left(\frac{1}{m}+\frac{m-1}{m}\rho_{xx}\right)\left(\frac{1}{m}+\frac{m-1}{m}\rho_{yy}\right)}}$$

### Appendix A.4. The Limit of ${\hat{\sigma }}_{2}^{2}$ under ${\bar{H}}_{2}$

The limit of ${\hat{\sigma }}_{2}^{2}=n^{-1}\sum_{i=1}^{n}{\left(x_{i}-y_{i}-\delta_{2}\right)}^{2}$ is $\sigma_{2}^{2}=E{\left(x_{i}-y_{i}-\delta_{2}\right)}^{2}$. Since $E\left(x_{i}-y_{i}-\delta_{2}\right)=p_{x}-p_{y}-\delta_{2}=0$ under ${\bar{H}}_{2}:p_{x}=p_{y}+\delta_{0}$, $E{\left(x_{i}-y_{i}-\delta_{2}\right)}^{2}=\mathrm{var}\left(x_{i}-y_{i}-\delta_{2}\right)=\mathrm{var}\left(x_{i}-y_{i}\right)=\mathrm{var}\left(x_{i}\right)+\mathrm{var}\left(y_{i}\right)-2\rho_{2}\sqrt{\mathrm{var}\left(x_{i}\right)\mathrm{var}\left(y_{i}\right)}$. Here, $\rho_{2}=\mathrm{corr}\left(x_{i},y_{i}\right)$,

$$\mathrm{var}\left(x_{i}\right)=\frac{1}{m^{2}}\left\{m\mathrm{var}\left(x_{i1}\right)+m\left(m-1\right)\mathrm{cov}\left(x_{i1},x_{i2}\right)\right\}$$

$$=\frac{\mathrm{var}(x_{i1})}{m}+\frac{m-1}{m}\rho_{xx}\mathrm{var}\left(x_{i1}\right))=p_{x}\left(1-p_{x}\right)\left(\frac{1}{m}+\frac{m-1}{m}\rho_{xx}\right)$$

and, similarly,

$$\mathrm{var}\left(y_{i}\right)=p_{y}\left(1-p_{y}\right)\left(\frac{1}{m}+\frac{m-1}{m}\rho_{yy}\right)$$

$$=\left(p_{x}-\delta_{0}\right)\left(1-p_{x}+\delta_{0}\right)\left(\frac{1}{m}+\frac{m-1}{m}\rho_{yy}\right)$$

since $x_{ij}∼\mathrm{Bernoulli}\left(p_{x}\right),y_{ij}∼\mathrm{Bernoulli}\left(p_{y}\right)$.
